# A miRNA screen procedure identifies *garz* as an essential factor in adult glia functions and validates *Drosophila* as a beneficial 3Rs model to study glial functions and GBF1 biology

**DOI:** 10.12688/f1000research.23154.2

**Published:** 2020-07-23

**Authors:** Catarina Gonçalves-Pimentel, David Mazaud, Benjamin Kottler, Sandra Proelss, Frank Hirth, Manolis Fanto

**Affiliations:** 1 Department of Basic and Clinical Neuroscience, King's College London, London, SE5 9NU, UK; 2 Champalimaud Research, Champalimaud Foundation, Av. Brasília, Lisbon, 1400-038, Portugal; 3 Institut du Cerveau et de la Moelle épinière (ICM), 47, bd de l'hôpital, Paris, F-75013, France

**Keywords:** miRNA, glia, Drosophila, screens, GBF1

## Abstract

Invertebrate glia performs most of the key functions controlled by mammalian glia in the nervous system and provides an ideal model for genetic studies of glial functions. To study the influence of adult glial cells in ageing we have performed a genetic screen in *Drosophila* using a collection of transgenic lines providing conditional expression of micro-RNAs (miRNAs). Here, we describe a methodological algorithm to identify and rank genes that are candidate to be targeted by miRNAs that shorten lifespan when expressed in adult glia. We have used four different databases for miRNA target prediction in *Drosophila* but find little agreement between them, overall. However, top candidate gene analysis shows potential to identify essential genes involved in adult glial functions. One example from our top candidates’ analysis is *gartenzwerg *( *garz*). We establish that *garz* is necessary in many glial cell types, that it affects motor behaviour and, at the sub-cellular level, is responsible for defects in cellular membranes, autophagy and mitochondria quality control. We also verify the remarkable conservation of functions between *garz* and its mammalian orthologue, GBF1, validating the use of *Drosophila* as an alternative 3Rs-beneficial model to knock-out mice for studying the biology of GBF1, potentially involved in human neurodegenerative diseases.

Research highlights **Scientific benefits:**

- This screen has provided a thorough analysis of glial functions in ageing
- Potential to shortcut gene discovery through miRNAs effect. The screening of only ~200 mutant lines potentially targets >6000 genes.
- Potential to identify complex regulatory networks that include miRNAs and target genes.
- Validated the identification of essential genes for the adult nervous system and their functions specifically in motor control.
- An open-access searchable database for future discoveries upon improved precision of miRNA-target predictions.

**3Rs benefits:**

- This screening method provides an alternative approach for studying genes important in glial biology, without the need for animal experiments.
- Example validation that *Drosophila* can be used to study the biology of GBF1, instead of *in vivo* vertebrate animal models, such as zebrafish or mouse.

**Practical benefits:**

- The searchable database can be easily updated upon emergence of updated miRNA target predictions.
- RNAi lines are publicly available from the Vienna Drosophila Resource Centre stock collection.
- Genetic studies in Drosophila are quicker and more sophisticated compared to vertebrate studies. They also maintain high conservation of functions.

**Current applications:**

- Uncovered the function of *garz* in glial cells for membrane trafficking, autophagy and mitochondria quality control.
- Study of genes, such as GBF1, involved in ageing and neurodegeneration

**Potential applications:**

- Identification of novel miRNA targets in glia
- Study of novel miRNA targets in glia
- Study of glial functions in controlling lifespan and healthspan

## Introduction

Despite the fact that glial cells were initially identified simply as the connective tissue of the brain ^1^, work developed in the past decades has shed a light on a much more intricate role for these cells in developing and maintaining nervous system homeostasis (reviewed in 2). From neuronal nutrient supply ^3^, to neurotransmitter recycling ^4– 6^, to being the first line of immune response in the brain ^7^, glial cells have been shown to actively contribute to the correct functioning of the brain.

More recently, several studies have been taking advantage of *Drosophila*’s powerful genetic manipulation to better understand the role of glia in the development and maintenance of the nervous system (see 8 for review).

The use of invertebrate models is also a powerful 3Rs solution to reduce and replace animal experiments. It expressly applies to complex matters in which cross-talk between different cell types (e.g. glia and neurons) is a focal point of the investigation, given that these complex environments are more difficult to model *in vitro* and *in silico*. Popular animal models for studying glial functions are zebrafish, which provide a useful platform for tissue and cell biology, with some capability for genetic manipulation ^9^ and genetically modified mice ^10^. Despite having a different developmental origin, glial cells have converged in *Drosophila* and mammals towards the same key functions of neurotransmission regulation, insulation and immune surveillance/phagocytosis ^8^, making the fruit-fly an organism of choice for studying the function of glial cells.

We have tackled the functions of glial cells in ageing. We have previously screened a large collection of miRNAs regarding their effects on *Drosophila’s* lifespan upon ectopic expression in glial cells in adult flies and have validated this screen through the analysis of *repo,* an already-established key glia gene ^11^. The experimental advantage of performing a miRNA-based screen followed by *in silico* identification and ranking of predicted miRNAs target transcripts ^11, 12^ has, however, its bottleneck in the validation of the action of the genes of interest. In principle, the specific knockdown of predicted target genes should mimic, to some extent, the phenotype obtained upon corresponding miRNA overexpression.

In fact, using databases of predicted miRNA-target genes previously allowed us to identify *repo* as an important player for maintaining glial function and, consequently, homeostasis in the adult brain ^11^. We have shown that while the *miR-1*- *repo* axis is physiologically relevant only in the embryo during the glia versus haemocyte cell fate choice ^13^, the miRNA-target relationship can be exploited as a discovery tool to identify the functions of a target gene in a different context, namely adult glial functions ^11^.

While the focus on *repo* was based on its already-established role in glia cell function, here we attempt a global and unbiased systematic *in silico* approach. In order to systematically identify potential target genes that could account for the lifespan phenotype, focusing on the miRNAs that shortened lifespan, we set out to devise a quantitative algorithm. The aim of this algorithm is to identify and rank the predicted target genes so that those ranking on top would be the most relevant for adult glia in lifespan and ageing.

This is followed by experimental validation of the function of these targets in adult glia in the same paradigm used in the miRNAs screen.

We conclude that this approach is valid but has issues of efficiency given the large number of predicted targets that do not recapitulate the expected phenotype. We also establish that there is no significant synergy generated by focusing on the common predictions between all available miRNAs target databases. Nevertheless, the main outcome of our work is a list of candidate genes whose function is essential in glial cells during ageing. These genes can be studied in the future in *Drosophila*, with the tools identified here, rather than in genetically modified mouse models or in zebrafish, providing an incentive towards animal replacement and reduction and advancing the 3Rs. Mouse and zebrafish neuroscientists and geneticists could take advantage of this information to test preliminary approaches and exploratory experiments in *Drosophila*, prior to validation in their system reducing the number of animals used. Alternatively, they may entirely replace vertebrate animals with *Drosophila* to study highly conserved genes and glial functions.

The success of this *in silico* approach is exemplified by our analysis of one of the top predicted targets: *gartenzwerg* ( *garz*), the fly orthologue of GBF1 ** (golgi brefeldin A resistant guanine nucleotide exchange factor 1), a small GTPase guanine exchange factor. Here, we show that *garz* is an essential factor in glia homeostasis maintenance.

Small GTPases regulate a wide range of cellular events such as proliferation, morphology, nuclear transport and vesicle formation ^14^. The conversion from GDP-bound (inactive) to GTP-bound (active) forms of these enzymes relies on the activity of GTPase activating proteins (GAPs) and guanine nucleotide exchange factors (GEFs). While GAPs are responsible for their inactivation through GTP hydrolysis, GEFs are responsible for their activation promoting the exchange of GDP by GTP ^15^.

GEFs belonging to the Sec7 domain protein family are responsible for the activation of Arf (ADP-ribosylation factor) GTPases which are associated with the recruitment of coat proteins (COP) to vesicle budding sites ^16– 18^. GBF1 is part of this family ^19^ and is highly conserved in all eukaryotes, conferring significant translatability of the findings obtained using different model organisms.

Strongly localized in the cis-Golgi compartment, GBF1 has been shown to regulate vesicle trafficking between the endoplasmic reticulum (ER) and the Golgi apparatus ^20– 24^. Mutated versions or knock-down of *garz* expression brings about epithelial morphogenesis defects during development conditioning embryonic trachea and larval salivary gland formation ^20, 21^. Additionally, in accordance with a role in membrane delivery and vesicular trafficking, silencing of *garz* in these glands impairs membrane delivery of adhesion molecules ^25^. Independently from its role in secretion, GBF1/ *garz* has also been implicated in pinocytosis ^26^; intestinal stem cell survival ^27^; cell cycle ^28, 29^; unfolded protein response events ^29^; mitochondria morphology and function ^30^; and autophagy ^31, 32^.

Here we show that *garz* knock-down resulted not only in lifespan reduction but also in motor deficits of adult flies and in subcellular phenotypes indicative of dysfunctions in trafficking, autophagy and mitochondria. Additionally, miRNAs overexpression and *garz* knockdown phenotypes were reverted by expression of its mammalian orthologue GBF1, stressing the conservation of functions and the appropriateness of using *Drosophila* in place of vertebrate models to study the biology of GBF1.

## Methods

### Online resources and in silico algorithms for target identification and ranking

The following databases were used for the prediction of miRNA targets:

- MicroCosm ( https://www.ebi.ac.uk/enright-srv/microcosm/htdocs/targets/v5/)
- microRNA.org ( http://www.microrna.org/microrna/home.do)
- TargetScan ( http://www.targetscan.org/fly_72/)
- PicTar ( https://pictar.mdc-berlin.de/)

Each of the databases provides for every miRNA a numerical prediction of the likelihood of targeting a given gene (Score). For MicroCosm and PicTar this was used without additional steps. In the case of miRNA.org this score is a negative value and we have squared it to obtain a positive number. In the case of TargetScan a numerical score was calculated on the basis of the information provided by the database as follows: conserved 8mer = 10 points, conserved 7mer-m8 = 6 points, conserved 7mer-1A = 4 points, poorly conserved 8mer = 8 points, poorly conserved 7mer-m8 = 4 points and poorly conserved 7mer-1A = 2 points. A detailed explanation of the 8mer and 7mer species can be found on the TargetScan website and in the original publication ^33^.

The algorithm for ranking targets within each database consists of two steps.

- Step 1 - column (Score)*Av(χ ^2^) or (Score ^2^)*Av(χ ^2^):
  For each miRNA, every target score (or its square value) was multiplied by the Average Chi square (χ ^2^) obtained in the miRNAs screen (from Table 1). Information regarding different mRNAs for the same gene, where available, was grouped under the same gene name
- Step 2 - column Σ(Score)*Av(χ ^2^)] or Σ(Score ^2^)*Av(χ ^2^)]:
  For each target gene, as defined by its CG number/accession ID, all values resulting from all miRNAs predicted to target the same gene were summed in a final ranking value. Information regarding different mRNAs from the same gene, where available, was grouped under the same gene name.

The algorithm for comparing the ranking between different databases and providing a final common ranking consists of two steps:

- Step1 - column Normalised Σ[(Score)*Av(χ ^2^)] or Normalised Σ[(Score ^2^)*Av(χ ^2^)]
  For each database the Σ(Score)*Av(χ ^2^)] was normalised to 100 and then weighted for the fraction of miRNAs present in the database, out of the total tested in our miRNAs screen. For TargetScan the groups of miRNAs families were counted as one unit in each case.
- Step 2 – column Σ{Normalised Σ[(Score ^(2)^)*Av(χ ^2^)]} For each target gene, all values from all databases were summed into a final ranking number.

**Table 1.** Average strength of miRNAs that shorten lifespan in adult glia. To determine the strength of miRNAs in our lifespan assay we have used the averaged χ ^2^ values from each transgenic line used in our previously published analysis ^11^. When only one line was tested for a given miRNA, the value was divided in half, i.e. assuming a neutral value of 0 for a second putative untested line. For the TargetScan database, some miRNAs are grouped in families requiring an amendment to our approach. In this case, we have averaged all miRNAs in the given families. Additionally, some of the lines tested for these grouped miRNAs had, in the original screen the opposite effect of what is here considered, i.e. extending lifespan with respect to the control used. To account for this opposite effect the χ ^2^ values for these miRNAs have been given negative values and have been effectively subtracted, when calculating the Av(χ ^2^) parameter.

miRNAs	Av(χi ^2^)	Av(χi ^2^) Targetscan
1	44.2375	
3	55.0050	23.8583 (3 + 309 + 318)
8	11.8700	
9a	94.4800	89.4917 (9a + 9b + 9c)
9b	106.5150	
9c	67.4800	
10	5.8250	
12	22.2150	
31	1.7233	
34	61.6733	
79	75.6650	
92a	95.6050	72.245 (92a + 92b + 310 + 312+ 313)
92b	28.3000	
124	82.5700	
133	48.0200	
137	42.7400	
184	27.4050	
193	47.5300	
219	2.1550	
263b	5.5650	
274	22.6400	
276b	41.4350	25.02 (276a + 276b)
277	24.5850	
278	77.6300	
279	13.5000	-4.104 (279 + 286 + 996)
287	2.2550	
310	102.4833	72.245 (92a + 92b + 310 + 312+ 313)
312	32.8850	
313	50.7100	
315	24.5900	
316	2.6950	
318	26.3850	
375	39.2500	
932	25.8700	
958	25.4200	
968	35.0700	
977	4.7065	
978	70.4600	
980	31.0400	
989	44.3800	
992	7.8050	
995	7.0500	2.695 (285 + 995 + 998)
999	2.7750	
1015	3.8550	

### Drosophila stocks and husbandry

Flies were kept on standard cornmeal agar food (0.8% w/v agar, 2% w/v cornmeal, 8% w/v glucose, 5% w/v Brewer’s yeast, 1.5% v/v ethanol, 0.22% v/v methyl- 4-hydroxybenzoate, 0.38% v/v propionic acid) at 18°C or room temperature. Unless stated otherwise, *w ^1118^* flies were used as control. The following lines were acquired from the Bloomington collection: *w ^1118^* (RRID:BDSC_3605), *repo-*Gal4 (RRID:BDSC_7415), *NP2222-*Gal4 (RRID:DGGR_112830), *moody-*Gal4, *elav-*Gal4 (RRID:BDSC_8765), *tub-*Gal80 ^ts^ (RRID:BDSC_7019). *alrm-*Gal4 (RRID:BDSC_67031) was kindly provided by M. Freeman (University of Massachusetts) ; *UAS-miR-1*, *UAS-miR-79* and *UAS-miR-315* were generated by E. Lai (Sloan Kettering Institute) for the miR library ^34^; *UAS-garz*-RNAi (42140/GD and 42141/GD) as well as all RNAi lines used are from Vienna *Drosophila* Resource Center (VDRC); *gliotactin-*Gal4 was provided by R. Sousa-Nunes; *UAS-mito*-GFP was provided by J. Bateman; *UAS-garz*; *UAS-garz ^Sec7-^*; *UAS-GBF1* and *UAS-ΔGBF1 ^Sec7-^* were kindly provided by S. Luschnig.

### Lifespan

Lifespan analysis was performed as previously described ^35^. Briefly, crosses were maintained at 18°C throughout the whole development of the progeny. Within the first 5 days post-eclosion, adult flies were collected, and equal numbers of female and male flies were pooled together. An equal number of flies was distributed in three vials, a total of 60 flies was used. This group size has a power of 0.8 in one tailed survival test at 50% survival for the control group and 29% for an experimental group at 0.05 significance. Lifespan assessment was performed in a controlled environment of 29°C and 60% humidity, three times a week. Upon short CO _2_ anaesthesia (5 s), the number of dead vs alive flies was counted, and the alive flies transferred into a fresh vial.

### Motor behaviour assay

Single fly tracking was carried out as previously described ^11^. In each experiment, up to 20 flies per genotype were placed into individual glass tubes. This group size has a power of 0.9 and significance 0.05 for three groups with an effect size of 0.48, as measured for the mean bout length. All the genotypes were positioned on the same platform, having two shaft-less motors placed underneath each subplatform containing each, one genotype. The protocol used consisted of 6 stimuli events equally split during a period of 2 h and 15 min, the first one starting after 30 min of recording and the last one 30 min before the end of the protocol. Each stimuli event was composed of 5 vibrations of 200 ms spaced by 500 ms. The x/y position of each single fly was tracked and analysed using DART software 1.0 (freely distributed upon request to info@bfklab.com) in order to evaluate the relative speed and activity before, during and after the stimuli event. The speed analysis was used for the “Stimuli Response Trace” and the general activity used to deduce “Active Speed”, “Mean Bout Length” and “Inter-Bout Interval”, using a custom-made modification of the DART software ^36^. Raw data were analysed with GraphPad Prism for statistical significance and DART-derived graphs were edited with Adobe Illustrator CC2017 (RRID:SCR_010279).

### Immunostaining

Flies (N=5–10) were briefly (5 s) anesthetized with CO _2_ and kept on ice, entire fly brains were dissected under a stereoscope and immediately fixed in 4% paraformaldehyde (PFA, from EMS) in Phosphate Buffer Saline (PBS) for 30 min. After washing with PBS, the brains were incubated for blocking in PBS with 0.3% triton-X (BDH 306324N) (PBT) and 10% foetal bovine serum (Sigma F4135) for 1 hr. Primary antibody incubation was done overnight at 4°C and followed by three washes (20 min each) in PBT. Secondary antibody incubation for 1hr at room temperature was followed by three washes. All steps were in 50-µl volume in a 96-well plate on a gentle rocker. Brains were then mounted on a slide in Vectashield with DAPI (Vector Labs). The following primary antibodies, diluted in blocking solution (see above): anti-Repo (1/100, mouse DSHB 8D12, RRID:AB_528448); anti-GFP (1/1000, rabbit, Life technologies, A11122) anti-GFP(1/100, mouse, Roche, RRID:AB_390913), anti-GFP (1/500, chicken, kindly provided by M. Meyer); anti-Ref(2)P (1/2000, rabbit, a gift of Tor Erik Rusten). Secondary antibodies were all from Life technologies (conjugated with Alexa-488, Alexa-555 or Alexa-666) and diluted 1/200 in blocking solution (see above).

Z-stacks at intervals of 0.3 µm or 5 µm were taken at 1024×1024 pixel/inch resolution. For control vs *garz ^IR^* comparisons, microscope settings were established using control flies to have a GFP signal below saturation and kept unchanged throughout all acquisitions. All images were acquired with a Leica TCS SP5 confocal microscope and mitochondria sphericity, volume and surface area in Figure 3B,C were measured using the 3D Object Counter 2.0.1 plugin ^37^ in the ImageJ Fiji 1.52n software (RRID:SCR_002285).

### Statistical analysis

All statistical analysis was performed with Graph- Pad Prism 7 software (RRID:SCR_002798). For all lifespans, the statistical analysis was performed using the log–rank test of the Kaplan and Meier method. For behavioural experiments (DART), the statistical analysis was done by one-way ANOVA using Dunnett’s multiple comparisons post hoc test. Significance is shown by asterisks in all figures as follows: *P<0.05, **P<0.01, ***P<0.001, and ****P<0.0001.

### Randomization and blinding

In each experiment the desired number of flies were selected haphazardly from a much larger cohort of flies with the same genotype and sex. Blinding was performed in lifespan and behaviour by masking the genotypes with a numerical or alphabetical serial labelling.

## Results

### Development of an algorithm for ranking miRNA target genes for their relevance in adult glia in lifespan and ageing

Firstly, such algorithm should prioritise the information for the miRNAs that had the strongest effect on the fly lifespan in our miRNA screen. To achieve this, we have quantified the average strength of each miRNA using the Chi square (χ ^2^) of each Kaplan Mayer analysis ( Table 1).

To identify potential target genes, we used four different databases available online: EBI MicroCosm, PicTar, microRNA.org and TargetScan. Each database weights the likelihood of every miRNA to target a given gene with a numerical score. Where this is different, for TargetScan, we calculated a numerical score on the basis of the sequence information provided by the database (see *Methods*).

Therefore, to rank target genes within each database taking into account both the likelihood of being targeted by a given miRNA and the strength of the effect of this miRNA in adult glia, we first multiplied the average strength of each miRNA from our screen (values in Table 1) by the strength of the target prediction (Score) given by the database, obtaining the parameter (Score)*Av(χ ^2^). This was done for all miRNAs tested in our screen that were present in each database.

Because a given gene can be targeted by more than one miRNA, to rank its overall importance in adult glia, we have summed all the values obtained for a given gene that were calculated for different miRNAs, obtaining the parameter Σ[(Score)*Av(χ ^2^)]. In the case of TargetScan, some miRNAs are grouped in families and we have considered them as a single unit value. This underweights these miRNAs in comparison to others and the genes targeted by them (for instance a gene targeted by miR-9a, miR-9b and miR-9c would obtain a Σ[(Score)*Av(χ ^2^)] that is the sum of three (Score)*Av(χ ^2^) in the other databases, but for TargetScan it would only reflect one (Score)*Av(χ ^2^). Our reasoning was that grouped miRNAs in TargetScan was not taking into account valuable information and this should be reflected in a penalisation in the ranking.

**Figure 1.**
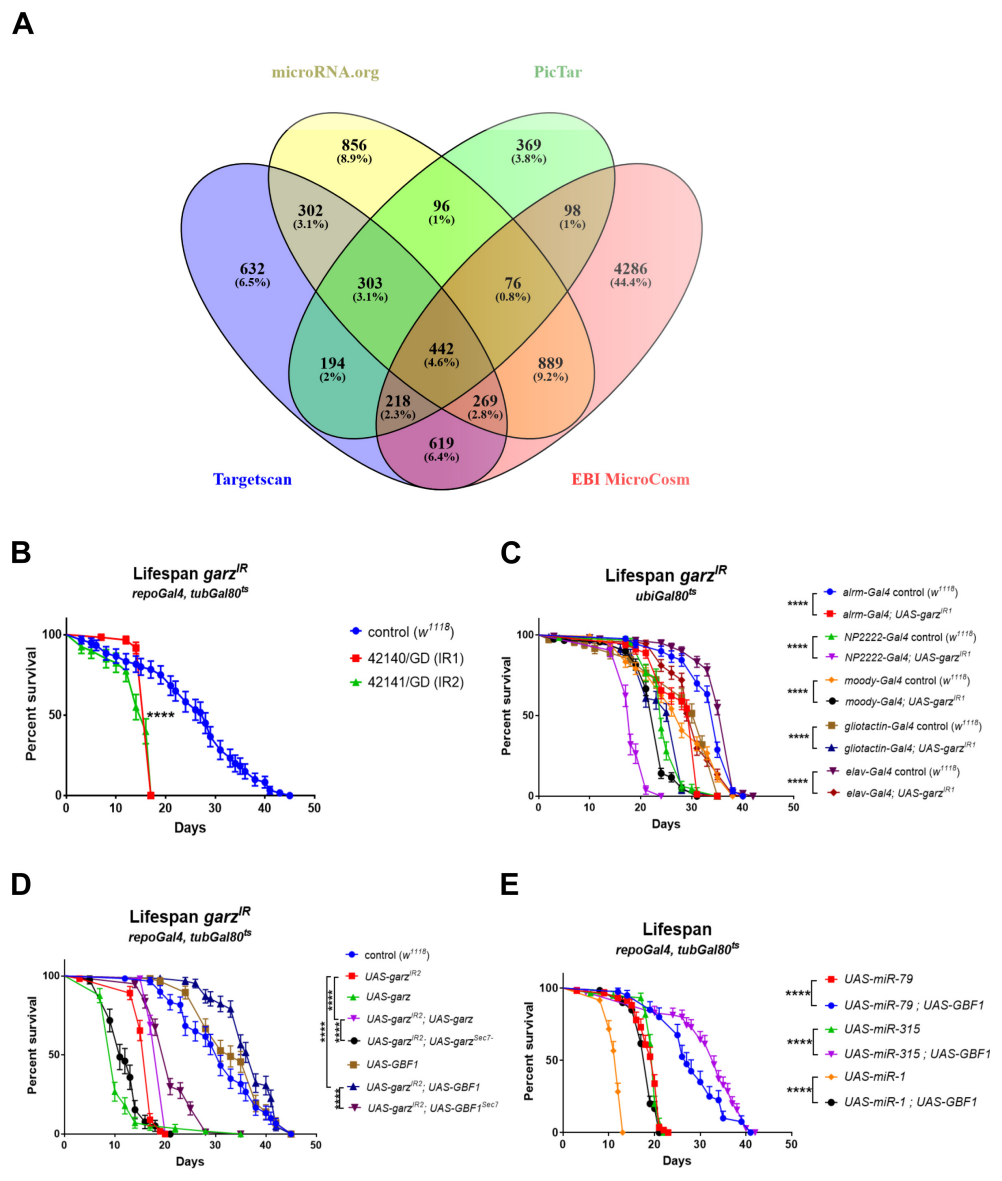
Effects of *garz* knock-down in adult glial cell. ( **A**) Venn diagram referring to the data in Table 2 and illustrating the overlap between the four different databases used to predict gene targets of the miRNAs whose expression in the adult glia resulted in a significant reduction in fly lifespan. Only 520 target genes are in common among all four databases, *garz* falls in this group. A remarkably large number of genes as targets were uniquely predicted by the MicroCosm database. ( **B**) Two RNAi lines against *garz* bring about a very significant reduction in fly lifespan in comparison to controls, when expressed in all adult glia. N=60 for each genotype, Error bars SEM, pairwise comparisons: Log-rank (Mantel-Cox) test. The full dataset can be accessed at DOI 10.17605/OSF.IO/8E3NS as part of Table 3. ( **C**) Knock-down of *garz* in sub-populations of glial cells, astrocyte-like ( *alrm-Gal4*), Cortex glia ( *NP2222-Gal4*), sub-perineural glia ( *moody-Gal4*), perineural and PNS glia ( *gliotactin-gal4*) or in neurons ( *elav-Gal4*) brings about a significant reduction in lifespan in comparison to controls. N=60 for each genotype, Error bars SEM, pairwise comparisons: Log-rank (Mantel-Cox) test. The full dataset can be accessed at DOI 10.17605/OSF.IO/HQCDG. ( **D**) Lifespan reduction due to RNAi against *garz* in adult glia is rescued by an exogenous *UAS-garz* transgene and by a transgene expressing the human orthologue GBF1 under UAS control. Note that overexpression of *garz* in an otherwise wt background is highly detrimental to fly lifespan, whereas overexpression of GBF1 in a wt background has no adverse effects. Mutations leading to a non-functional Sec7 domain eliminate or drastically reduce the ability of *garz* or *GBF1* transgenes to rescue fly lifespan. N=60 for each genotype, Error bars SEM, pairwise comparisons: Log-rank (Mantel-Cox) test. The full dataset can be accessed at DOI 10.17605/OSF.IO/5RGEF. ( **F**) Co-expression of human GBF1 significantly extends the short lifespan caused by overexpression of miR-1, miR-79 and miR-315 in adult glia. N=60 for each genotype, Error bars SEM, pairwise comparisons: Log-rank (Mantel-Cox) test. The full dataset can be accessed at DOI 10.17605/OSF.IO/B37DF.

In conclusion we have ranked target genes according to Σ[(Score)*Av(χ ^2^)] for EBI MicroCosm ( *Extended data* Table 1) ^38^, PicTar ( *Extended data* Table 2) ^38^, microRNA.org ( *Extended data* Table 3) ^38^ and TargetScan ( *Extended data* Table 4) ^38^. Surprisingly, this revealed that there was very little agreement among the four databases. The top-ranking genes obtained using the same algorithm were very different and only 5.6% (i.e. 520 genes) of target predictions were common to all four databases ( Figure 1A).

To rank these common targets for their predicted overall relevance in adult glia in ageing, we have devised additional steps. First, to make the numerical rankings from each database comparable, we have calculated the Normalised Σ[(Score)*Av(χ ^2^)] parameter by normalising the maximum value to 100. Additionally, we have weighted this number for the fraction of miRNAs present in each database, out of the total tested in our miRNAs screen. Out of 44 miRNAs screened, 31 were present in EBI Microscosm, 28 in PicTar, 43 in microRNA.org and 40 in TargetScan. The rationale for this weighting was to prioritise the databases carrying more information that was relevant to our screen. Then, for each target gene, we have combined all these scores from the four databases generating the final parameter Σ{Normalised Σ[(Score ^(2)^)*Av(χ ^2^)]} for all targets, including the 520 that were commonly predicted by all databases ( Table 2).

**Table 2.** Identification and ranking of target predictions common to all four databases. The ranking scores from all four databases were pooled to obtain a global rank of all targets predicted by our analysis and a list of targets that are predicted by all four databases. Because the different databases contained information about some, but not all, miRNAs analysed in our screen we have weighted the completeness of each database by normalising the Σ[(Score)*Av(χ ^2^)] by the fraction of miRNAs listed in the database, out of the ones tested in our screen. In addition, to make the ranking from each database equally valued in this analysis, we have normalised each score to 100 as a maximum possible value for each database – column Normalised Σ[(Score)*Av(χ ^2^)]. Thereafter, all values for each target have been added – column Σ{Normalised Σ[(Score)*Av(χ ^2^)]} – for each target and for a specific list of 520 targets that have been predicted by all four databases, albeit with different scores. Only the top 30 rows are shown here. The full table can be accessed at https://doi.org/10.17605/OSF.IO/QWUAY.

Pooled Non Redundant			520 Common elements in "Targetscan", "microRNA.org", "PicTar" and "EBI MicroCosm":		
GENE NAME	CG No	Σ{Normalised Σ[(Score ^(2)^)*Av(χi ^2^)]}	GENE NAME	CG No	Σ{Normalised Σ[(Score ^(2)^)*Av(χi ^2^)]}
CG7852	CG7852	206.8084	CG7852	CG7852	206.8084
CrebA	CG7450	165.9009	CadN	CG7100	148.5861
CadN	CG7100	148.5861	nerfin-1	CG13906	129.7977
sha	CG13209	133.6386	Cpr50Ca	CG13338	125.1063
CG31191	CG31191	130.1998	Nak	CG10637	103.4518
nerfin-1	CG13906	129.7977	CG11206	CG11206	95.6901
CG13338	CG13338	125.1063	CG8128	CG8128	94.2880
CG4297	CG4297	122.1155	up	CG7107	94.0563
Mef2	CG1429	113.9984	CG3077	CG3077	0.0000
Khc-73	CG8183	110.5961	porin	CG6647	89.8516
A2bp1	CG32062	108.8272	CG14015	CG14015	89.2605
Nak	CG10637	103.4518	CG33090	CG33090	82.8792
Rbp9	CG3151	102.6802	ttk	CG1856	81.3790
CG11206	CG11206	95.6901	Sbf	CG6939	73.9455
sinu	CG10624	94.9798	Klp68D	CG7293	72.5983
CG8128	CG8128	94.2880	CG12024	CG12024	72.3875
up	CG7107	94.0563	CG10737	CG10737	71.3749
w	CG5123	91.9243	rau	CG8965	70.9517
CG3077	CG3077	91.2297	CG9426	CG9426	69.5509
porin	CG6647	89.8516	salm	CG6464	68.5351
CG14015	CG14015	89.2605	Thd1	CG1981	66.7116
CG14274	CG14274	87.6812	Dysb	CG6856	65.1018
Eip93F	CG18389	84.3128	rho	CG1004	64.6470
CG33090	CG33090	82.8792	Vha68-1	CG12403	64.4111
lola	CG12052	81.8661	CG8323	CG8323	64.1433
ttk	CG1856	81.3790	CG4853	CG4853	63.3083
srp	CG3992	81.2668	CG9650	CG9650	62.1572
ck	CG7595	80.9393	SP555	CG14041	61.7540
CG32767	CG32767	78.9667	CdsA	CG7962	61.6698
sdk	CG5227	74.9601	RhoGAP68F	CG6811	61.4035
Sbf	CG6939	73.9455	Opa1	CG8479	61.2329

**Table 3.** Match between experimental RNAi and predictions for targets common to all databases. Strength of the effect on fly lifespan of RNAi lines against some of the gene targets predicted by all four databases. For most genes two different RNAi lines have been tested. In red are the lines that, in agreement with the miRNA prediction, shorten the lifespan, in comparison to controls ( *w ^1118^*) when specifically expressed in the adult flies with *repo-Gal4* and *tub-Gal80 ^ts^*. In green are the lines that have the opposite effect and prolong lifespan. In black are the lines that had no effect. Highlighted in yellow are the genes for which all lines tested had the same effect and shortened lifespan. Highlighted in pink is the gene for which all lines tested had the same effect and prolonged the lifespan. To combine the target RNAi strength with the strength of the prediction we have averaged the χ² for each miRNA according to the same rules followed in Table 1 and multiplied it for the final score from Table 2 – column Σ{Normalised Σ[(Score)*Av(χ²)] *{Av(χ²)IR}. The top rank was achieved by *garz*, which was also one of the 6 genes with all RNAi lines tested having the same effect. We have also repeated the same procedure separately for the four different databases using the final normalised score (Normalised Σ[(Score)*Av(χ²)]) obtained from each database in Tables 2, 3, 4 and 5, and also reported in Table 2– columns Normalised Σ[(Score)*Av(χ²)] *{Av(χ²)IR} for each database. The predicting power for the combination of all databases and for each database sums all scores in this column to quantify the global predicting value of each database in comparison to their combination. This is further normalised by the number of predicted targets for the whole screen, to measure the efficiency of each database when predicting target genes. The full dataset can be accessed at DOI 10.17605/OSF.IO/8E3NS.

Common to all databases								EBI MicroCosm		PicTar		microRNA.org		Targetscan	
Target	Lines	Median survival	χi ^2^	p-values	Av(χi ^2^)IR	Σ {Normalised Σ[(Score)*Av(χi ^2^)]}	Σ {Normalised Σ[(Score)*Av(χi ^2^)]} *{Av(χi ^2^)IR}	Normalised Σ[(Score)*Av(χi ^2^)]	Normalised Σ[(Score)*Av(χi ^2^)] *{Av(χi ^2^)IR}	Normalised Σ[(Score)*Av(χi ^2^)]	Normalised Σ[(Score)*Av(χi ^2^)] *{Av(χi ^2^)IR}	Normalised Σ[(Score)*Av(χi ^2^)]	Normalised Σ[(Score)*Av(χi ^2^)] *{Av(χi ^2^)IR}	Normalised Σ[(Score)*Av(χi ^2^)]	Normalised Σ[(Score)*Av(χi ^2^)] *{Av(χi ^2^)IR}
Blimp-1	108374/KK	27	3.9090	0.0480	2.1158	49.9482	105.6803	15.8908	33.6217	3.4973	7.3996	14.1685	29.9777	16.3916	34.6813
	34978/GD	33	0.3226	0.5700											
Bx	106495/KK	24	4.1660	0.0412	5.7270	58.9239	337.4571	12.5711	71.9946	10.0509	57.5614	21.5938	123.6680	14.7081	84.2331
	2971/GD	27	7.2880	0.0069											
CadN	101644/KK	31	0.0939	0.7593	-2.1481	148.5861	-319.1725	20.0306	-43.0271	46.4332	-99.7416	38.4779	-82.6531	43.6443	-93.7507
	1092/GD	31	4.3900	0.0361											
CG10737	106383/KK	32	0.1800	0.6714	18.2600	71.3749	1303.3054	35.5040	648.3023	5.7567	105.1168	13.7227	250.5759	16.3916	299.3104
	8996/GD	19	36.3400	<0.0001											
CG11206	42943/GD	35	8.4800	<0.0001	-3.7402	95.6901	-357.9003	9.0084	-33.6933	8.5247	-31.8840	34.9136	-130.5839	43.2434	-161.7391
	42945/GD	28	0.9996	0.3174											
CG12918	105503/KK	35	10.6900	0.0011	-5.3436	52.1314	-278.5678	20.8109	-111.2046	3.3907	-18.1183	18.0949	-96.6912	9.8350	-52.5538
	38082/GD	31	0.0029	0.9573											
CG13606	109997/KK	31	3.1620	0.0754	1.6099	52.6384	84.7431	14.5549	23.4320	4.7616	7.6658	16.6199	26.7566	16.7020	26.8887
	45481/GD	29	0.0578	0.81											
CG3077	109618/KK	31	3.1620	0.0754	1.6012	91.2297	146.0771	23.2887	37.2898	6.4559	10.3372	41.8153	66.9546	19.6699	31.4955
	25630/GD	29	0.0404	0.8301											
CG32105	108747/KK	28	2.5080	0.1132	2.5570	43.3198	110.7687	10.3112	26.3658	2.2363	5.7182	20.9373	53.5367	9.8350	25.1480
	51267/GD	28.5	2.6060	0.1064											
CG33090	23407/GD	29	0.5904	0.4423	0.3904	82.8792	32.3560	21.1135	8.2427	4.0624	1.5859	16.7976	6.5578	40.9057	15.9696
	28033/GD	28	0.1904	0.6626											
CG3376	12226/GD	29	0.3743	0.5406	0.1872	42.0182	7.8637	8.3405	1.5609	5.9978	1.1225	17.8450	3.3397	9.8350	1.8406
CG3534	109666/KK	35	8.7280	0.0031	-4.3640	48.6526	-212.3199	17.7977	-77.6690	3.4824	-15.1971	17.5376	-76.5341	9.8350	-42.9197
	41276/GD	30	4.522E-07	0.9995											
CG3624	36304/GD	27	0.4244	0.5148	3.1832	55.4638	176.5522	16.9653	54.0040	2.6852	8.5476	23.3858	74.4417	12.4274	39.5589
	956/GD	26	5.9420	0.0148											
CG4360	105000/KK	25	2.6370	0.1044	4.7780	47.9093	228.9108	18.0506	86.2459	2.4584	11.7464	11.0087	52.5996	16.3916	78.3190
	26520/GD	25	6.9190	0.0085											
CG4984	10057/GD	31	0.4919	0.4831	-2.9691	59.4745	-176.5827	18.0490	-53.5884	7.6279	-22.6477	7.6745	-22.7861	26.1230	-77.5605
	107854/KK	33	6.4300	0.0112											
CG5599	106456/KK	29	0.9585	0.3276	22.6593	40.5821	919.5590	8.8509	200.5549	1.0127	22.9470	12.8686	291.5939	17.8498	404.4632
	16505/GD	18	44.3600	<0.0001											
CG6129	110171/KK	23	12.2400	0.0005	14.1150	41.6798	588.3099	6.8082	96.0981	2.8427	40.1250	14.9821	211.4727	17.0467	240.6141
	22094/GD	21	15.9900	<0.0001											
CG7510	105469/KK	31	1.4210	0.2333	6.9305	49.2790	341.5279	14.8554	102.9554	2.4046	16.6651	12.5453	86.9454	19.4736	134.9621
	8532/GD	24	12.4400	0.0004											
CG8121	105866/KK	33	5.6770	0.0172	-0.7240	54.1836	-39.2289	7.7300	-5.5965	2.9878	-2.1632	8.4767	-6.1371	34.9891	-25.3321
	43952/GD	30	2.2380	0.1347											
	43953/GD	31	1.2670	0.2603											
CG8128	107574/KK	31	0.0685	0.7936	-8.1508	94.2880	-768.5205	19.9651	-162.7308	3.0917	-25.1994	54.8397	-446.9861	16.3916	-133.6042
	97740/GD	34.5	16.3700	<0.0001											
CG8303	107101/KK	28	7.3890	0.0066	11.0345	49.7826	549.3262	15.8126	174.4840	3.3083	36.5052	14.2702	157.4640	16.3916	180.8730
	4918/GD	25	14.6800	0.0001											
CG8323	4861/GD	29	3.5680	0.0589	1.7840	64.1433	114.4316	18.4807	32.9695	4.3704	7.7968	13.6601	24.3696	27.6321	49.2957
CG8360	23461/GD	34	4.8070	0.0283	-2.4035	56.7583	-136.4185	16.8753	-40.5598	5.7226	-13.7542	11.2122	-26.9484	22.9482	-55.1561
CG8417	106461/KK	38	23.1700	<0.0001	-11.5805	52.5854	-608.9652	15.8653	-183.7277	4.3959	-50.9072	15.9326	-184.5075	16.3916	-189.8227
	49509/GD	31	0.0090	0.9244											
CG9376	106062/KK	34	20.1300	<0.0001	-10.0650	15.8820	-159.8522	7.2495	-72.9661	0.5563	-5.5996	3.1890	-32.0968	4.8872	-49.1895
CG9650	104402/KK	33	0.8672	0.3517	0.4336	62.1572	26.9522	7.6917	3.3352	7.0478	3.0560	23.3065	10.1060	24.1113	10.4549
	23170/GD	29.5	2.53E-05	0.996											
Cpr	107422/KK	38	31.0800	<0.0001	-15.5400	47.5051	-738.2286	14.3186	-222.5113	9.1431	-142.0838	14.2084	-220.7984	9.8350	-152.8352
Dysb	106957/KK	32	1.6390	0.2005	1.6958	65.1018	110.3974	19.4330	32.9539	4.5585	7.7301	20.0348	33.9743	21.0755	35.7391
	34354/GD	29	0.1023	0.7491											
	34355/GD	34	3.3460	0.0674											
endoB	104712/KK	38	39.1400	<0.0001	-16.4445	24.5428	-403.5939	0.1061	-1.7449	6.1164	-100.5814	7.4080	-121.8216	10.9122	-179.4460
	29291/GD	24	6.2510	0.0124											
Ero1L	11045/KK	31	0.0187	0.8912	3.1058	45.3898	140.9738	14.8674	46.1758	5.0706	15.7486	15.6169	48.5035	9.8350	30.5458
	51169/GD	25	6.1930	0.0128											
garz	42140/GD	17	42.4800	<0.0001	45.3800	31.3660	1423.3876	4.7120	213.8301	0.5962	27.0568	6.4735	293.7671	19.5843	888.7336
	42141/GD	16	48.2800	<0.0001											
Gfat2	105129/KK	31	0.9625	0.3265	0.9978	41.9788	41.8843	15.8440	15.8083	3.1358	3.1287	13.1641	13.1345	9.8350	9.8128
	17187/GD	27	1.0330	0.3094											
Myd88	106198/KK	31	1.7490	0.186	1.9035	6.4706	12.3167	4.1638	7.9258	0.0121	0.0231	2.0501	3.9023	0.2446	0.4656
	25402/GD	28	2.0580	0.1515											
Nak	109507/KK	29	0.4342	0.5099	0.2233	103.4518	23.1023	19.0669	4.2579	16.3947	3.6612	36.5532	8.1629	31.4370	7.0203
	35482/GD	29	0.0124	0.9112											
pdm2	102126/KK	31	2.1180	0.1455	1.0590	25.7957	27.3176	6.5096	6.8937	0.9607	1.0173	4.8823	5.1704	13.4431	14.2362
porin	101336/KK	38	15.4000	<0.0001	-7.7000	89.8516	-691.8569	62.5375	-481.5385	2.5203	-19.4065	2.9915	-23.0343	21.8023	-167.8776
Ptp69D	27091/GD	27	2.4770	0.1155	1.4233	52.3718	74.5408	14.2880	20.3361	6.8889	9.8050	19.0121	27.0599	12.1828	17.3398
	40631/GD	29	0.3696	0.5432											
RASSF8	105823/KK	31	0.9831	0.3214	0.8924	34.0327	30.3690	4.4412	3.9631	0.7073	0.6312	8.3362	7.4388	20.5480	18.3360
	26520/GD	27	0.8016	0.3706											
raw	101255/KK	35	17.5900	<0.0001	10.6350	43.2677	460.1515	11.3523	120.7318	5.8320	62.0234	15.1805	161.4449	10.9028	115.9513
	24532/GD	14	38.8600	<0.0001											
regucalcin	105509/KK	31	1.0220	0.312	9.8860	56.0884	554.4895	15.5743	153.9677	15.7528	155.7325	14.9263	147.5610	9.8350	97.2283
	39945/GD	22	18.7500	<0.0001											
RhoGAP68F	107775/KK	28	3.0870	0.0789	1.5819	61.4035	97.1323	14.2428	22.5303	1.8217	2.8817	19.0121	30.0747	26.3269	41.6457
	34520/GD	31	0.0767	0.7818											
Sbf	22317/GD	35	8.9180	0.0028	-4.4590	73.9455	-329.7229	28.9451	-129.0661	4.3431	-19.3659	17.0058	-75.8288	23.6515	-105.4621
sens	106028/KK	34	7.399	0.0065	-3.6995	49.4627	-182.9872	14.7634	-54.6171	2.6482	-9.7972	8.9293	-33.0340	23.1217	-85.5388
Sirt2	103790/KK	30	5.7360	0.0166	-4.8680	44.5340	-216.7917	15.6316	-76.0947	5.3448	-26.0184	13.7227	-66.8020	9.8350	-47.8766
	21999/GD	31	4.0000	0.0455											
SP555	39821/GD	19	19.5700	<0.0001	9.7850	61.7540	604.2626	20.8951	204.4589	7.8086	76.4074	17.9622	175.7604	15.0880	147.6358
T48	100334/KK	11	21.3000	<0.0001	10.6500	49.6111	528.3581	4.3790	46.6361	1.1091	11.8117	10.2365	109.0189	33.8865	360.8914
Thd1	110439/KK	29	0.7217	0.3956	0.3609	66.7116	24.0729	19.9882	7.2127	2.1996	0.7937	24.2192	8.7395	20.3047	7.3269
Tm1	34119/GD	19	10.7400	0.001	5.3700	37.4083	200.8823	9.7281	52.2401	3.4708	18.6380	9.6231	51.6761	14.5862	78.3281
toe	107893/KK	23	15.4400	<0.0001	13.0900	47.8611	626.5024	17.7450	232.2815	3.2504	42.5476	17.0308	222.9338	9.8350	128.7395
	46515/GD	27	10.7400	0.001											
up	27853/GD	25	7.8600	0.0051	3.9300	94.0563	369.6411	32.8545	129.1183	24.6085	96.7114	24.4104	95.9330	12.1828	47.8784
Vha68-1	17102/GD	28	0.7032	0.4017	0.6250	64.4111	40.2570	9.5403	5.9627	3.4957	2.1848	26.8446	16.7779	24.5305	15.3316
	46397/GD	28	0.5468	0.4596											
**Predicting Power**						**4843.1509**		**1178.4057**		**279.9651**		**1284.1503**		**2100.6298**	
**Predicting power normalised for number of targets predicted**						**0.3227**		**0.1704**		**0.1556**		**0.3902**		**0.6990**	

**Table 4.** Match between experimental RNAi and predictions for targets not in common to all databases. Strength of the effect on fly lifespan of RNAi lines against some of the gene targets predicted by some, but not all, databases. In red are the lines that, in agreement with the miRNA prediction, shorten the lifespan, in comparison to controls ( *w ^1118^*) when specifically expressed in the adult flies with *repo-Gal4* and *tub-Gal80 ^ts^*. In green are the lines that have the opposite effect and prolong lifespan. In black are the lines that had no effect. Highlighted in yellow is the gene for which all lines tested had the same effect and shortened lifespan. To combine the target RNAi strength with the strength of the prediction we have averaged the χ² for each miRNA according to the same rules followed in Table 1 and multiplied it for the final normalised score (Normalised Σ[(Score)*Av(χ²)]) obtained from each database in *Extended data* Tables 1, 2, 3and 4 [49], and also reported in Table 2 – columns Normalised Σ[(Score)*Av(χ²)] *{Av(χ²)IR} for each database. The predicting power for each database sums all scores in this column to quantify the global predicting value of each database. This is further normalised by the number of predicted targets for the whole screen, to measure the efficiency of each database when predicting target genes, as in Table 3. The full dataset can be accessed at DOI 10.17605/OSF.IO/QTASN.

						EBI MicroCosm		PicTar		microRNA.org		Targetscan	
Target	Lines	Median survival	χi ^2^	p-values	Av(χi ^2^)IR	Normalised Σ[(Score)*Av(χi ^2^)]	Normalised Σ[(Score)*Av(χi ^2^)] *{Av(χi ^2^)IR}	Normalised Σ[(Score)*Av(χi ^2^)]	Normalised Σ[(Score)*Av(χi ^2^)] *{Av(χi ^2^)IR}	Normalised Σ[(Score)*Av(χi ^2^)]	Normalised Σ[(Score)*Av(χi ^2^)] *{Av(χi ^2^)IR}	Normalised Σ[(Score)*Av(χi ^2^)]	Normalised Σ[(Score)*Av(χi ^2^)] *{Av(χi ^2^)IR}
CG15544	39997/GD	28	0.2505	0.6167	0.1253	1.2446	0.1559	0.0137	0.0017			0.1564	0.0196
CG1623	107655/KK	26	3.0500	0.0808	2.9325	14.6090	42.8407			20.3535	59.6866	13.6183	39.9357
	32665/GD	27	2.8150	0.0934									
CG17712	105119/KK	31	0.3146	0.5749	4.5853	16.4402	75.3831			12.5865	57.7130	9.8350	45.0962
	32987/GD	26	8.8560	0.0029									
CG3678	26267/GD	22	35.9900	<0.0001	24.6750	22.2661	549.4160			15.6470	386.0907	9.8350	242.6775
	49793/GD	24	13.3600	0.0003									
CG4893	110188/KK	31	1.0850	0.2977	5.8825	19.6009	115.3024			21.6375	127.2824	20.3047	119.4422
	22356/GD	26	10.6800	0.0011									
fray	101058/KK	38	22.8600	<0.0001	-11.2926							1.0625	-11.9986
	27944/GD	31	0.2749	0.6001									
Ggamma1	28894/GD	32.5	3.4180	0.0645	1.7090			10.0845	17.2344	3.4702	5.9305	18.7955	32.1215
inx2	102194/KK	33	0.2422	0.6226	0.1211	0.0986	0.0119			10.2664	1.2433		
Nek2	103408/KK	33	0.6786	0.4101	3.1163	29.1504	90.8415			13.6972	42.6847	9.8350	30.6487
	40052/GD	26	5.5540	0.0184									
nord	39901/GD	31	0.0068	0.9341	0.0034			0.0120	0.0000	3.5694	0.0122	0.3669	0.0013
Paf-AHalpha	101683/KK	33	6.2970	0.0121	-3.1485	2.4176	-7.6118						
Pif1B	49782/GD	17	36.0700	<0.0001	18.0350	8.5626	154.4266			5.5158	99.4768	18.7342	337.8716
sna	50003/GD	32	3.0820	0.0791	2.1454	0.5810	1.2464			0.3121	0.6696	6.6337	14.2319
	50004/GD	33	0.1852	0.6670									
	6232/GD	31.5	3.1690	0.0751									
tor	101154/KK	31	2.5570	0.1098	1.5355	16.4688	25.2879			11.3850	17.4817	16.3916	25.1693
	36280/GD	31	0.5140	0.4734									
**Predicting Power**						**1047.3007**		**17.2361**		**798.2716**		**875.2168**	
**Predicting power normalised for number of targets predicted**						**0.1515**		**0.0096**		**0.2426**		**0.2913**	

### Systematic experimental testing of the prediction, ranking and effectiveness of different databases

To test these predictions, we decided to screen for the lifespan effect, a number of RNAi lines from Vienna *Drosophila* Resource Center (VDRC) that were already present in our stock collection. These corresponded to a random selection of approximately 10% (51 out of 520) of commonly predicted target genes. Adopting a similar strategy used for the miRNA screen, we have used the *repo-Gal4, tub-Gal80* ^ts^ inducible system to trigger the RNAi expression in all glial cells in adult flies. As negative control, we used the offspring of crossing *repo-Gal4, tub-Gal80* ^ts **^ to *w ^1118^* throughout the screen. The expectation was that RNAi against these target genes in adult glia, would phenocopy the effect of the miRNAs that are predicted to target them, therefore shortening lifespan.

The gold standard commonly used by the *Drosophila* community to gain confidence about the effects of RNAi knock-down is to obtain a similar effect when testing two RNAi lines against the same gene (2-RNAi lines criterion). Remarkably, only in six cases at least two different RNAi lines tested for the same gene delivered the shorter lifespan phenotype that was predicted ( Table 3). In another case both RNAi lines tested had the same effect, but it was the opposite of the predicted one, extending lifespan with respect to the control flies.

In other cases (11/51) there was an overall confirmation of the prediction, but the two RNAi lines tested for one given target did not share the same effect or we were able to test only one line. The largest group (19/51) was made by cases in which there was no effect and surprisingly in a remarkable number of cases (14/51) there was an overall effect opposite to that predicted, albeit either the two RNAi lines tested for one given target did not share the same effect or we were able to test only one line.

In addition to the 2-RNAi lines criterion we have devised a quantitative index for ranking these targets by combining their effect in the RNAi screen (averaging the Chi square for the RNAi lines targeting each gene, Av(χ ^2^)IR) with the strength of the prediction in all combined databases (Σ{Normalised Σ[(Score)*Av(χ ^2^)]}).

This parameter (Σ{Normalised Σ[(Score)*Av(χ ^2^)]}*{Av(χ ^2^)IR}) highlighted *garz*, one of the six targets satisfying the 2-RNAi lines criterion, as the top target ( Table 3). However, there was incomplete agreement with respect to the rest of the ranking between the two criteria, i.e. our scoring system and the rule of 2-RNAi lines, with only four of the ten top scores coming from target genes satisfying the 2-RNAi lines criterion.

We also tested 14 additional targets that were differentially predicted by the different databases. We were able to further identify five targets that confirmed the predicted phenotype, one satisfying also the 2-RNAi lines criterion, while two had the opposite overall effect ( Table 4).

A comparison between these two groups, the common to all databases and the differentially predicted, highlights that the fraction of validated prediction is similar, but the chance of finding false positives (i.e. targets that had the opposite effect to that predicted) is paradoxically higher in the commonly predicted group (15/51 in the common and 2/14 in the differential).

Considering the lack of tangible benefits of focusing on the commonalities between the different databases, we have then exploited our validation analysis to quantify the prediction capability of each of the four databases to identify the most valid for our screen. For all targets tested, both from the common group ( Table 3) and from the differential group ( Table 4), we have calculated the database-specific Normalised Σ[(Score)*Av(χ ^2^)] *{Av(χ ^2^)IR} parameter by combining the quantification of the lifespan effect of the RNAi lines (average Chi square in the RNAi screen) with the normalised predicted score from each database. Then, to rank databases we have summed all these results (with a negative value for false positives) to determine the predicting power score. TargetScan had the highest predicting power for the list of common targets, while MicroCosm had the highest capacity for target identification among the differential targets. PicTar had the lowest predicting power in all cases. However, MicroCosm also predicted the largest number of genes as targets of our miRNA screen, with over 44% of them not shared by the other databases. We reasoned that this lack of efficiency in EBI Microcosm had to be considered and when normalising for the total number of predicted targets from each database, as a measure of the predicting power efficiency, TargetScan showed a greater efficiency in both cases, followed by miRNA.org.

### Fly lifespan and motor behaviour are affected by *garz* knockdown in adult glia

As mentioned, we ranked the target genes from the RNAi confirmed predictions and decided to further investigate the top ranked target, *garz*, the fly orthologue for *GBF1* ^19, 20, 39^).

Pan-glial knockdown of *garz* with *repo-Gal4* specifically during adulthood strongly reduced lifespan. This was true for both RNAi lines tested when compared to *w ^1118^* median lifespan control ( Figure 1B). Different glial cell types present in the adult fly brain have specific morphology and function ^40^. In order to test if a specific glial sub-population could account for the observed phenotype, we targeted the knockdown of *garz* using established Gal4 driver lines: astrocyte-like ( *alrm-Gal4*), cortex ( *NP2222-Gal4*), subperineural ( *moody-Gal4*) and peripheral ( *gliotactin-Gal4*) glia. In all sup-populations tested, the downregulation of *garz* caused a reduction in lifespan, albeit not as strong as the pan-glial knockdown ( Figure 1C). This suggests that a combination of multiple functions is affected by *garz*.

We also analysed the effects of pan-neuronal ( *elav-Gal4*) knockdown of *garz*. This also led to a significant shortening of lifespan although the effect was milder than the one obtained with pan-glial *garz* knockdown ( Figure 1B vs 1C), either because of differences in Gal4 line strength or because of a higher impact of *garz* function in glial cells for maintenance of the brain homeostasis.

We then focused on rescuing the glia-related shorter lifespan phenotype using exogenous transgenes for *garz* and human *GBF1*. Although the overexpression of *garz* alone in adult glia had a very toxic effect, when combined with the *garz-*RNAi overexpression, promoted a modest but significant rescue of the lifespan ( Figure 1D). This suggests that *garz* levels need to be tightly controlled in the fly. On the other hand, overexpression of the human *GBF1* was entirely neutral for fly lifespan when expressed on its own and fully rescued the lifespan phenotype when co-expressed with *garz* RNAi. This indicates a remarkable conservation in functions between *garz* and *GBF1*.

For both garz and GBF1, the presence of a functional Sec7 domain, which is responsible for the catalytic activity of GEF proteins domain ^24^, was important to exercise their rescue activity ( Figure 1D). In the case of *UAS-garz*, a mutation of the Sec7 domain entirely eliminated the rescue of *garz* knock down, actually aggravating toxicity. This also indicates that the toxicity of *garz* overexpression is not dependent on the catalytic GEF function of *garz*, possibly suggesting a dominant negative effect by sequestration of binding partners in catalytically inactive complexes. Additionally, in the case of *UAS-GBF1*, the rescue effect was significantly reduced, albeit not entirely eliminated, by an inactive Sec7 domain ( Figure 1D).

Human GBF1 showed a remarkable capability to fully rescue lifespan shortening upon *garz* knockdown in glia. We then asked whether it would also be able to rescue the lifespan shortening induced by miRNAs predicted to target *garz*. From our database analysis, miR-1, miR-79 and miR-315, all causing a strong reduction of lifespan ^11^, were among the miRNAs predicted to target *garz* and may be rescued by *GBF1*. Indeed, *UAS-GBF1* was able to significantly rescue the phenotypes caused by the overexpression of these miRNAs in glia ( Figure 1E). *GBF1* co-overexpression was able to rescue the lifespan for miR-79 and miR-315 to what would be commonly observed in wild-type flies. These results confirm our initial predictions and establish *garz* as the main mediator of the effect on lifespan caused by overexpression of miR-79 and mir-315 in adult glia. The partial rescue of the miR-1 phenotype indicates that *garz* is only partially responsible for the effect of miR-1 in adult glia and is in accordance with the previously reported role of *repo* in miR-1-mediated lifespan shortening ^11^.

**Figure 2.**
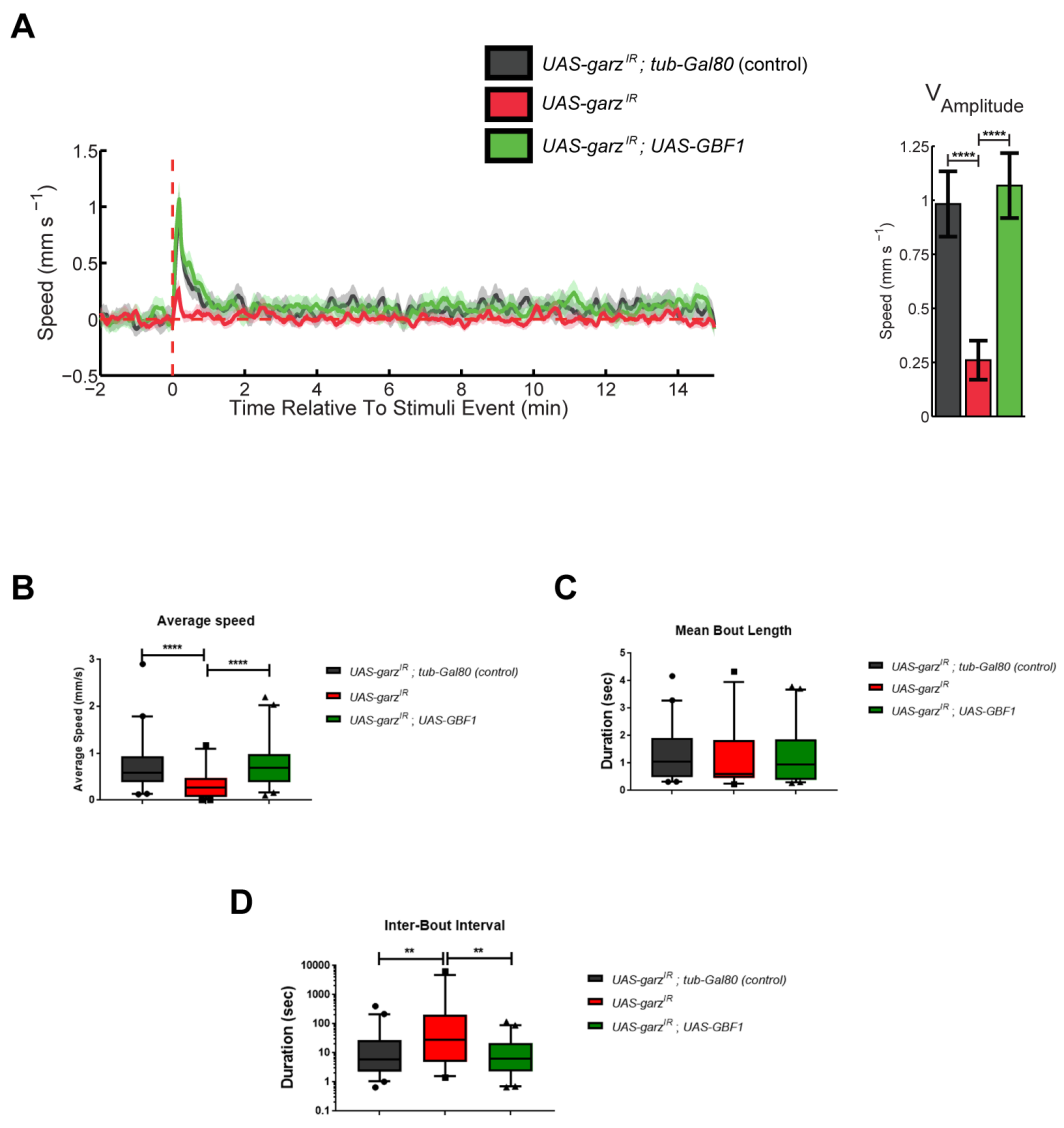
Knock down of *garz* in adult glial cells leads to significant impairment of fly motor functions. All data in this figure represent a grouping of two independent experiments with a total number of flies analysed (N) of 35–40. Error bars represent SEM in all graphs. Untreated track data can be accessed at DOI 10.17605/OSF.IO/UNJX7. ( **A**) Stimulus response curve for control flies (black), *garz* RNAi (red) and co-expression of GBF1 and *garz* RNAi (green). The graph is an average of 6 tracks for each of the stimuli received at 15 min intervals (See Methods). All genotypes also include *repo-Gal4* and *ubi-Gal80 ^ts^* to express the transgenes in all adult glia. In control flies the presence of *tub-Gal80* blocks any expression of UAS-transgenes. The graph to the right reports the mean amplitude of the response to a train of stimuli, which is significantly reduced by RNAi against *garz*, and this reduction is reverted to normal level by co-expression of human GBF1. One-way ANOVA, Dunnett’s multiple comparisons post hoc test. ( **B**) Average speed analysis of the same flies as in A. RNAi against *garz* significantly slows down fly motility and this is rescued by human GBF1. One-way ANOVA, Dunnett’s multiple comparisons post hoc test. ( **C**) Mean bout length analysis of the same flies as in A. No significant difference is detected in this parameter. One-way ANOVA, Dunnett’s multiple comparisons post hoc test. ( **D**) Mean interbout interval analysis of the same flies as in A. RNAi against *garz* significantly increases the time spent in inactivity by flies and this is rescued by human GBF1. One-way ANOVA, Dunnett’s multiple comparisons post hoc test.

We have previously described an automated unbiased and high-throughput method to analyse fly motor activity ^11^). When using this paradigm, we unravelled an impact of glial *garz* knockdown on the amplitude of the response to a train of stimuli and GBF1 co-overexpression rescued this response ( Figure 2A). When looking at spontaneous activity parameters, i.e. non-stimulus driven, in the same experiment, flies expressing *garz*-RNAi showed a reduced average speed and an increased interval between bouts of movement without reflecting in the overall bout movement duration. Both average speed and inter-bout interval were fully rescued by the co-overexpression of GBF1 ( Figure 2B–D). This analysis indicates that *garz* knock down affects not only lifespan but also the healthspan and motor activity both exogenously stimulated and internally generated, making flies slower and also pausing more.

### Subcellular effects of *garz* knockdown in adult glia

We next set out to determine the effects *garz* knock down had inside the glial cells that would correlate with behavioural and lifespan dysfunctions.

**Figure 3.**
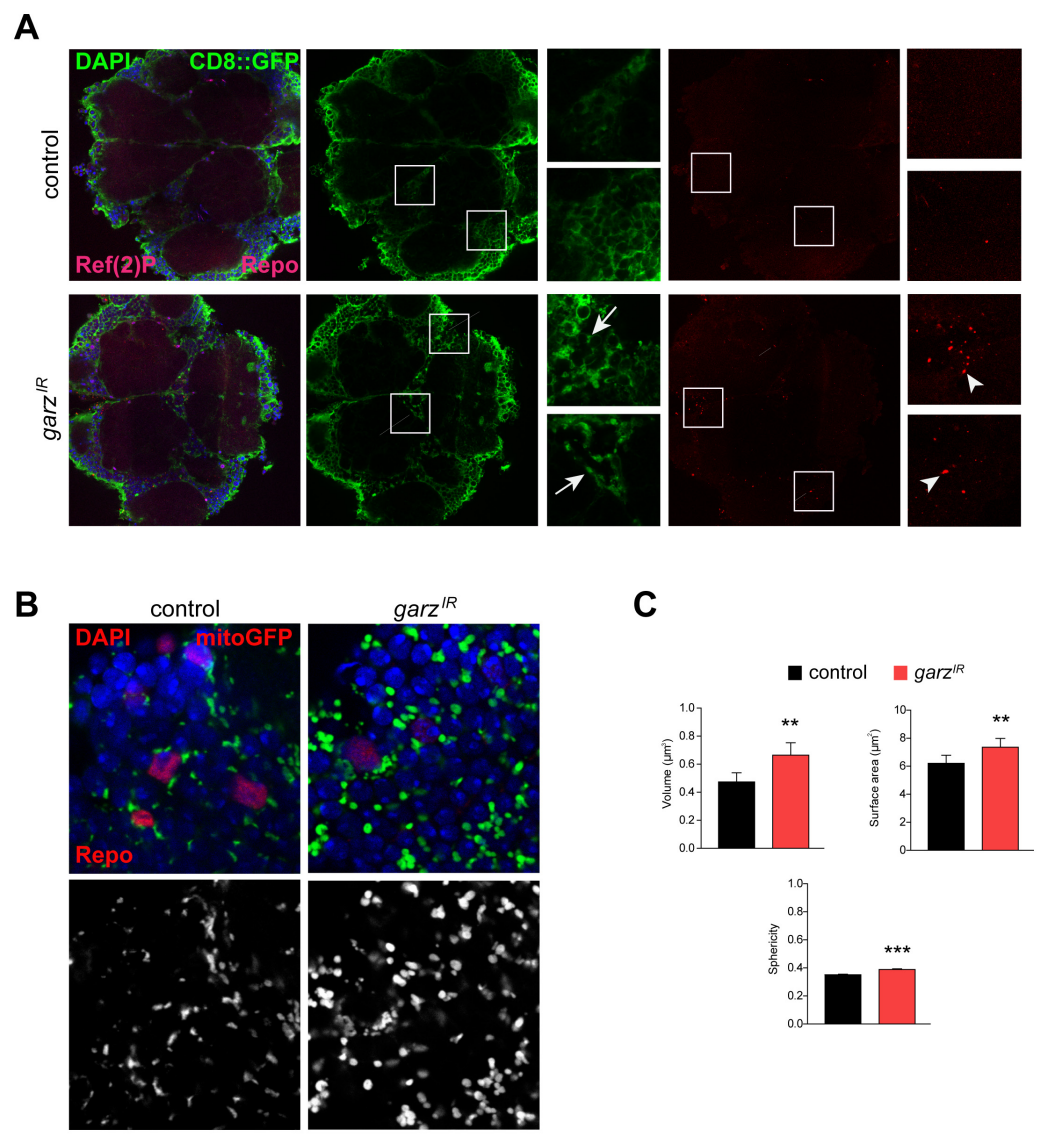
Sub-cellular dysfunctions caused by *garz* knock-down in adult glial cell. ( **A**) Representative single confocal sections of adult fly brains stained for DAPI (blue), GFP (green), Repo (magenta) and Ref (2)P (red). Pan glial knock-down of *garz* with *repo-Gal4* and *ubi-Gal80 ^ts^* leads to abnormal distribution of the plasma membrane targeted CD8-GFP protein (expressed from a *UAS-CD8-GFP* transgene in all glial cells) leading to gaps and blebs (arrows, see also Videao1 and 2), and to accumulation of Ref(2)P puncta (arrowheads). The full dataset can be accessed at DOI 10.17605/OSF.IO/96TS3. ( **B**) Representative single confocal section of adult fly brains stained for DAPI (blue), GFP (green) and Repo (red). The GFP signal also in back and white (lower panels) is due to the presence of a *UAS-mitoGFP* transgenes and detects mitochondria. ( **C**) Quantification of mitochondria parameters based on the GFP signal in B. Pan glial knock-down of *garz* with *repo-Gal4* and *ubi-Gal80 ^ts^* leads to significant increases in the volume, surface area and sphericity of mitochondria. Mann-Whitney non-parametric test. N=300 objects, randomly selected from 4 brains. Error bars represent SEM. The full dataset can be accessed at DOI 10.17605/OSF.IO/EXMTG.

It has been reported that *garz* knockdown impairs vesicle transport and membrane delivery during fly development ^25^. Thus, we analysed membrane distribution in the presence of *garz*-RNAi in adult brains. Driving the expression *CD8*- *GFP* in glia showed aberrant membrane distribution upon *garz* knockdown when compared to a more homogeneous distribution of the GFP signal in glia from control brains ( Figure3A, Videos 1 and 2). Such data suggests that overall membrane trafficking in glia may be impaired although we have not been able to detect failure in membrane delivery of the cell adhesion cadherin molecule CadN, whose potential glia localisation effects may, however, be masked by the unaffected CadN localisation in neurons, where CadN is highly expressed (data not shown, the full dataset can be accessed at DOI 10.17605/OSF.IO/7HRZS).

Video 1. CD8-GFP in control brains. 3D reconstruction of confocal stacks imaging of panglial (*repo-Gal4*) CD8-GFP expression in a control brain. Note the smooth appearance of the glial membranes highlighted by the green signal. The full dataset can be accessed at DOI 10.17605/OSF.IO/96TS3. Click here for additional data file. Copyright: © 2020 Gonçalves-Pimentel C et al. 2020

Video 2. CD8-GFP in garz knock-down brains. 3D reconstruction of confocal stacks imaging of panglial (*repo-Gal4*) CD8-GFP expression in a *garz* knock-down brain. Note the clustering and blebs formed by the glial membranes highlighted by the green signal. The full dataset can be accessed at DOI 10.17605/OSF.IO/96TS3. Click here for additional data file. Copyright: © 2020 Gonçalves-Pimentel C et al. 2020

Conflicting *in vitro* data has been reported for the effects of GFB1 ^31^ and *garz* ^32^ in what concerns autophagy regulation. Looking at the distribution of the Ref(2)p (the orthologue of mammalian p62) autophagy receptor ^43^ revealed Ref(2)p accumulation in puncta, suggesting a potential block in autophagic clearance in glial cells ( Figure 3A).

Finally, it has been suggested a role for GBF1 in the regulation of mitochondria morphology and function in yeast, *C. elegans* muscle and HeLa cells ^30^. Using *mito-GFP* transgene we were able to identify mitochondrial morphology defects in adult glial cells ( Figure 3B, C). Quantification of the main morphological parameters has unravelled an overall increased mitochondrial volume, surface and sphericity upon *garz* knockdown. These parameters may indicate a defect in mitochondria quality control and are in agreement with an impaired autophagic clearance, which has the potential to also affect mitophagy.

*Underlying data* contains the raw data behind these results ^44^.

## Discussion

We have previously screened a library of miRNAs for effects on *Drosophila’s* lifespan when expressed in adult glia and already established that this strategy can identify factors important for nervous system health in adult life ^11^. We aimed here at developing a generalizable global approach that would allow to identify the key target genes that mediate the actions of miRNAs in a given context. Focusing on miRNAs that shortened the lifespan, we devised an *in-silico* strategy to unravel a potential list of genes relevant for glial function and consequently brain homeostasis in adult flies. The outcome of this strategy had efficiency issues and highlighted the little overlap in the predictions made on the basis of four different databases for miRNA target prediction in *Drosophila.*

To put to the test the outcome of these *in silico* predictions, we have silenced individual genes by inducing the expression of specific RNAi in adult glia. The assumption being that RNAi downregulation of the top target genes would phenocopy the effect observed when expressing the miRNAs targeting them, i.e. lifespan reduction. Overall, however, the number of genes that, upon knockdown, reduced lifespan was remarkably low, and we could observe no tangible benefit of focusing on predictions in common to all four databases, versus targets differentially predicted in the different databases. It was also evident from our analysis that, among the databases, TargetScan and miRNA.org were considerably more efficient in delivering predictions that withstood the RNAi tests.

Therefore, the benefits of using miRNAs-based screens and *in silico* identification of targets, in place of much larger screens based on targeting single genes, have to be carefully evaluated and *in silico* selection of target genes should be based primarily on the TargetScan and miRNA.org databases. Nevertheless, the fraction of validated positive target genes by two criteria (7/65) and by at least one (22/65) is much larger than what usually expected in siRNA screens and suggests a 3/5-fold enrichment in positive hits. Thus, our method makes *Drosophila* screens a more appealing platform with reduced workload in comparison to traditional single gene targeted screens, whether by RNAi or genomic mutagenesis. This may have 3Rs benefits, facilitating the use of *Drosophila* as a model for preliminary studies on the genetic factors that influence a given biomedical process.

Our screen has also highlighted a number of genes that are strong, and in most cases unexpected, candidates for essential functions in adult glia in ageing. This list of genes provides a useful tool for scientists studying glial functions in ageing. In particular, all identified genes that have been validated by two RNAi lines have clear mammalian orthologues. *Drosophila* can therefore be used to study in detail the functions of these genes in the adult glial cells, in place of genetically modified mouse models.

To validate our findings, we focused on the top target of the genes commonly predicted by all databases and also by TargetScan, i.e. *garz*, the fly orthologue of the human GBF1.

The analysis of *garz* confirmed that this gene is absolutely required in adult glia, and also in neurons, for fly survival. Using our automated behavioural set up we could also establish that *garz* is essential in glia for locomotor activity in response to a stimulus or endogenously generated. Analysing the effects of silencing *garz* in different glial sub-populations showed that the strong reduction in lifespan could not be accounted for by one specific type of glia but rather due to a combined effect of silencing *garz* in all glial cells simultaneously, indicating that *garz* is essential for any glial cell type.

Our subcellular analysis suggests that the locomotor and lifespan defects correlate and possibly originate from a number of cellular defects in protein trafficking, autophagy and mitochondria quality control.

In *Drosophila*, mutated versions or knockdown of *garz* resulted in developmental epithelial morphogenesis defects ^20, 21^ and impaired membrane delivery of adhesion molecules ^25^. We have been able to identify membrane defects in glial membrane distribution, although not all membrane proteins seemed to be affected by *garz* knockdown. *garz* and *GBF1* have been identified as a positive autophagy regulator in *Drosophila* primary cultured muscle cells ^32^ and mammalian cells ^31^. An accumulation of Ref(2)P upon *garz*-RNAi expression in adult glia suggests an autophagic clearance deficits, in agreement with these studies.

*GBF1*-RNAi has been shown to affect mitochondrial morphology and function ^30^. Chemical inhibition of GBF1 in mammalian cells also showed condensed mitochondria and mislocalisation in the cell ^45^. Although mislocalisation of mitochondria is difficult to assess due to glial cell morphology in the *Drosophila* brain, *garz*-RNAi strongly affected mitochondria morphology suggesting a more condensed state which may be a reflection of an unbalanced fission/fusion regulation and mitochondria quality control ^46^.

Our analysis further suggested that there was remarkable functional conservation between *garz* and human GBF1. While the lack of toxicity of GBF1 overexpression, in comparison to Garz, may indicate some divergence and lack of dominant negative activity, this may also be due to different levels of expression or tags. Nevertheless, GBF1 was able to fully rescue, partially in a Sec7-domain dependent manner, the shorter lifespan and motor behaviour phenotypes caused by the silencing of *garz*. GBF1 was also able to rescue the lifespan shortening by three different miRNAs, miR-1, miR-79 and miR-315, validating that in our screen their effect is at least partially, and in some cases almost entirely, due to downregulation of *garz*.

Thus, these data validate both the logic and principles of miRNA screens, despite inefficiencies, and the use of *Drosophila* as a valid organism to study the biology of *garz*/ *GBF1*.

The identification of major cellular events regulated by *garz*/ *GBF1* ^27– 29, 47– 49^ has targeted such molecules for health and disease studies ^18^. Recently, it has been shown that siRNA knockdown of GBF1 causes intracellular Amyloid Precursor Protein (APP) accumulation in primary cortical neurons; overexpression of GBF1 contributes to APP trafficking and is dependent on its GEF activity ^50^. Inhibition of GBF1 with brefeldin A was also shown to lead to a new form of cellular degeneration and death in neurodegenerative diseases, based on destruction of the nuclear lamina ^51^.

Gbf1 conditional mutant mice have been generated in the Wellcome Trust Sanger Institute and are being phenotyped by the International Mouse Phenotyping Consortium ( https://www.mousephenotype.org/data/genes/MGI:1861607). We demonstrate here that *Drosophila* would constitute an ideal organism to put forward 3Rs-compliant alternatives and, at least partially, replace this mouse line in studies aiming at understanding the role of GBF1 in health and disease.

## Data availability

### Underlying data

Open Science Framework: miRNA-garz. https://doi.org/10.17605/OSF.IO/A5ZST ^44^.

This project contains the following underlying data:

- Table 2 (XLSX). (The complete Table 2.)
- Table 2 Data – Pimental *et al.,* 2020 (XLSX). (Data underlying Table 2.)
- Table 3 Data – Pimental *et al.,* 2020 (XLSX). (Data underlying Table 3.)
- Table 4 Data – Pimental *et al.,* 2020 (XLSX). (Data underlying Table 4.)
- Figure 1C Data - Pimentel *et al.,* 2020 (XLSX). (Data underlying Figure 1C.)
- Figure 1D Data - Pimentel *et al.,* 2020 (XLSX). (Data underlying Figure 1D.)
- Figure 1E Data - Pimentel *et al.,* 2020 (XLSX). (Data underlying Figure 1E.)
- Figure 2 Data - Pimentel *et al.,* 2020 (XLSX). (Data underlying Figure 2.)
- Figure 3A and videos. (TIFF images and ZIP files containing data underlying Figure 3A.)
- Figure 3B-C. (ZIP files containing raw images underlying Figure 3B, C.)
- Extended data Table 1- Data - Pimentel *et al.,* 2020 (XLSX). (Data underlying *Extended data* Table 1.)
- Extended data Table 2- Data - Pimentel *et al.,* 2020 (XLSX). (Data underlying *Extended data* Table 2.)
- Extended data Table 3- Data - Pimentel *et al.,* 2020 (XLSX). (Data underlying *Extended data* Table 3.)
- Extended data Table 4- Data - Pimentel *et al.,* 2020 (XLSX). (Data underlying *Extended data* Table 4.)
- Data not shown. (ZIP files containing images of membrane delivery of the cell adhesion cadherin molecule CadN.)

### Extended data

Open Science Framework: miRNA-garz. https://doi.org/10.17605/OSF.IO/K5HW9 ^38^.

This project contains the following extended data:

- **Extended Data Table 1. MicroCosm target prediction and ranking tables.** For each miRNA, ranking of target prediction - column (Score)*Av(χ ^2^) - was made by multiplying the Average χ ^2^ obtained in the screen (from Table 1) by the Score predicted in the MicroCosm database. In the total table, all values from a given target, resulting from all miRNAs were summed in a final ranking value in column Σ(Score)*Av(χ ^2^)]. This table and the full dataset can be accessed at DOI 10.17605/OSF.IO/R3ZX9.
- **Extended data Table 2. PicTar target prediction and ranking tables.** For each miRNA, ranking of target prediction - column (Score)*Av(χ ^2^) - was made by multiplying the Average χ ^2^ obtained in the screen (from Table 1) by the Score predicted in the PicTar database. In the total table, all values from a given target, resulting from all miRNAs were summed in a final ranking value in column Σ(Score)*Av(χ ^2^)]. This table and the full dataset can be accessed at DOI 10.17605/OSF.IO/MDKHR.
- **Extended data Table 3. miRNA.org target prediction and ranking tables.** For each miRNA, ranking of target prediction - column (Score ^2^)*Av(χ ^2^) - was made by multiplying the Average χ ^2^ obtained in the screen (from Table 1) by the square value of Score predicted in the miRNA.org database. The square value was used in this case as the scoring system used by miRNA.org delivers negative values, differently from the other databases. In the total table, all values from a given target, resulting from all miRNAs were summed in a final ranking value in column Σ(Score ^2^)*Av(χ ^2^)]. This table and the full dataset can be accessed at DOI 10.17605/OSF.IO/539J8.
- **Extended data Table 4. TargetScan target prediction and ranking tables.** The TargetScan database does not provide a scoring system for its predictions, rather a list of 8mer or 7mer sequences matched by the miRNA on the target and an information on the conservation of these sequences. We have attributed a numerical score to these sequences privileging the importance of 8mer vs 7mer and of conservation according to the scheme described in the Methods section. For each miRNA, ranking of target prediction - column (Score)*Av(χ ^2^) - was made by multiplying the Average χ ^2^ obtained in the screen (from Table 1, some values specifically generated averaging all miRNA grouped in a single family by TargetScan) by the Score obtained according to our above-mentioned scheme. In the total table, all values from a given target, resulting from all miRNAs were summed in a final ranking value in column Σ(Score)*Av(χ ^2^)]. This table and the full dataset can be accessed at DOI 10.17605/OSF.IO/WD6ZR.

Data are available under the terms of the Creative Commons Attribution 4.0 International license (CC-BY 4.0).
