# Supplementary material for: A miRNA screen procedure identifies garz as an essential factor in adult glia functions and validates Drosophila as a beneficial 3Rs model to study glial functions and GBF1 biology
Source: F1000Res. 2020 Jul 23;9:317. Originally published 2020 May 1. [Version 2] doi: 10.12688/f1000research.23154.2 (PMC7309417; doi:10.12688/f1000research.23154.2)
Supplement: Peer review history

# Peer review: Reviewer response for version 1

**DOI:** 10.5256/f1000research.25558.r63653

**Published:** 2020-06-22

**Authors:** Jeff W. Barclay

**Affiliations:** 1 Department of Cellular and Molecular Physiology, Institute of Translational Medicine, University of Liverpool, Liverpool, UK

This manuscript uses *Drosophila* to screen by miRNAs for essential glial genes, identifying and briefly investigating one of the candidates ( *gartenzweg* or *garz*). The screen appears to be conducted well, the data logically presented and the outcomes interesting and novel. The screening method and methodological algorithm is straight-forward and described well. I have no negative issues with the experiments and believe this is a valid topic for publication that will prove useful to the community. In addition, I agree whole-heartedly that the 3Rs benefits are well demonstrated for studying miRNAs, glia and GBF1 biology specifically.

 I have only minor suggestions for improvement, where the authors may want to reconsider wording to accurately reflect outcomes.

1. The overall interpretations of the manuscript are that this screen is identifying glial functions. From the manuscript, however, it is not clear what these functions actually are? The experiments manipulate expression in glial cells – and then measure broad outcome phenotypes such as lifespan and locomotion. However, I don’t think the authors are necessarily suggesting that the function of glia is lifespan or locomotion.  Later there are experiments that infer potential alterations to intracellular trafficking in glia, but I find these experiments more representative of *garz* function in glia rather than glial function itself.
2. The glia are targeted by miRNA expression and RNAi in the glia themselves. As miRNAs can be potentially released by exocytosis, it would be worthwhile to discuss the possibility for effects originating in other cells. The authors have done a nice control with the RNAi in neurons; however, this has partially replicated the glial RNAi effects and thus overall may reflect some transcellular effects.
3. The manuscript indicates remarkable conservation in function between *garz* and GBF1; however, I would suggest tempering that conclusion given that they do have divergent effects (e.g. one is toxic, one is not).
4. It isn’t entirely clear why CD8 trafficking was selected for investigation over other possibilities. Given the lack of effect on cadherin trafficking, the speculation that overall trafficking in glia is impaired seems premature.

Is the rationale for developing the new method (or application) clearly explained?

Yes

Are the 3Rs implications of the work described accurately?

Yes

Is the description of the method technically sound?

Yes

If applicable, is the statistical analysis and its interpretation appropriate?

Yes

Are the conclusions about the method and its performance adequately supported by the findings presented in the article?

Yes

If any results are presented, are all the source data underlying the results available to ensure full reproducibility?

Yes

Are a suitable application and appropriate end-users identified?

Yes

Are sufficient details provided to allow replication of the method development and its use by others?

Yes

Reviewer Expertise:

Invertebrate models, genetics, neuroscience. Referee suggested by the NC3Rs for their scientific expertise and experience in assessing 3Rs impact.

I confirm that I have read this submission and believe that I have an appropriate level of expertise to confirm that it is of an acceptable scientific standard.

# Response

**Published:** 2020-07-18

**Authors:** Manolis Fanto

**Affiliations:** King's College London, UK

We thank both reviewer for their competent assessment of our manuscript. We have now provided a revised version of our paper and reply here to the questions and the issues raised by both reviewers.

*1. The overall interpretations of the manuscript are that this screen is identifying glial functions. From the manuscript, however, it is not clear what these functions actually are? The experiments manipulate expression in glial cells – and then measure broad outcome phenotypes such as lifespan and locomotion. However, I don’t think the authors are necessarily suggesting that the function of glia is lifespan or locomotion.  Later there are experiments that infer potential alterations to intracellular trafficking in glia, but I find these experiments more representative of garz function in glia rather than glial function itself.*

 We understand the point of the reviewer. To clarify, we start from the assumption that affecting glial functions (i.e. insulation, trophism, phagocytosis and neuronal activity modulation) will impact the functionality of the nervous system, hence it will affect lifespan and behaviour. This is the assumption of our screen, which indirectly addresses the functions of glias. Similarly, we find that any alterations at the subcellular level reveal a function for *garz* in adult glial cells, exactly as the reviewer points out. The defects in autophagy, protein localization and mitochondria are likely to underlie the defects in glial functions, but we are not focusing here on the precise way in which glial functions are affected, rather on the validation of the screen via identification of *garz* as an essential molecule in adult glia.

*2. The glia are targeted by miRNA expression and RNAi in the glia themselves. As miRNAs can be potentially released by exocytosis, it would be worthwhile to discuss the possibility for effects originating in other cells. The authors have done a nice control with the RNAi in neurons; however, this has partially replicated the glial RNAi effects and thus overall may reflect some transcellular effects.*

 The reviewer is correct that miRNAs can be released by exocytosis, however it is still unclear what the overall impact of this release (and uptake) is, when looking at the global organism level. While in plants and in *C. elegans* the transcellular effect of miRNA is well documented to be a potent effector, this appears to be much more limited in *Drosophila* and mammals. With respect to Garz, the simplest explanation is that this ubiquitous Golgi protein is necessary also in neurons, and, as such, it will affect lifespan also from neurons.

*3. The manuscript indicates remarkable conservation in function between garz and GBF1; however, I would suggest tempering that conclusion given that they do have divergent effects (e.g. one is toxic, one is not).*

 While we agree with this observation toxicity levels may be due to:

1. Different expression levels.
2. Epitope tag: GFP-Garz vs HA-GBF1.
3. Dominant negative effect of Garz but not of GBF1.

We agree that explanation 3 would be in line with some difference between GBF1 and Garz and have slightly modified our text in the discussion to account for this effect. We still think that the conservation of effect between Garz and GBF1 is remarkable in consideration of the other two possibilities and of the rescue effects. While a negative result (lack of toxicity) could be attributed to several reason, the remarkable rescue obtained by GBF1 can be explained with a high level of confidence with GBF1 performing Garz functions.

*4. It isn’t entirely clear why CD8 trafficking was selected for investigation over other possibilities. Given the lack of effect on cadherin trafficking, the speculation that overall trafficking in glia is impaired seems premature.*

 Using CD8::GFP as a general membrane reporter we aimed at checking overall intracellular membrane trafficking effects specifically within glia. CadN is expressed in many but not all glial cells and it is much more widely expressed in neurons. In this sense any defect in glial cell distribution of CadN might have been masked by the largely normal distribution of CadN in neurons. We have further clarified this point in our manuscript and apologize for not having made that clearer in our first version.

# Peer review: Reviewer response for version 1

**DOI:** 10.5256/f1000research.25558.r62978

**Published:** 2020-06-01

**Authors:** Ivana Bjedov

**Affiliations:** 1 UCL Cancer Institute, University College London, London, UK

This manuscript by Gonçalves-Pimentel et al. describes an impressive series of very elegant and demanding longevity experiments used to develop an innovative methodological algorithm to identify and rank candidate genes that are targeted by miRNAs and that shorten lifespan when downregulated in adult glial cells. The work presented offers a comprehensive comparison of different miRNA target databases and link those to the longevity analysis screen. Advantage of miRNA is targeting multiple genes at once. For instance by screening 200 miRNA lines, this examines effect of down-regulation of approximately 6000 genes. However this subsequently presents a challenge to determine which genes are targeted with a particular miRNA and which gene is accountable for a given phenotype. Therefore, here, Gonçalves-Pimentel et al. produce an algorithm in which they link their miRNA lifespan screen results to multiple miRNA databases, with the final aim to rank genes that are predicted targets by these miRNA and that affect lifespan and ageing of the glial cells. Briefly, within each data base, each target score was multiplied by values of the lifespan screen results, and then ranking value for each target gene obtain for different data bases. Values obtained from all miRNA databases for each gene were summed for final ranking. This approach combined the effect of the miRNA on lifespan with the prediction of a gene being targeted by particular miRNA, using a variety of databases to strengthen the approach. Combining different databases is particularly important given a surprising difference in their target prediction.

 The top candidate from the screen is a gene *garz*, (mammalian orthologue of GBF1), for which they predict that its down-regulation in the glia shorten lifespan. Advantage of such approach is that it offers possibility for screening in an invertebrate organism to uncover genes potentially important in mammalian glia.

*garz* is a target for three miRNA, *miRNA-1*, *miRNA-79*, *and miRNA-315*. The authors carefully examine its affect in different population of glial cells, replicate shorter lifespan using two different *garz *RNAi lines, overexpress human GBF1 to rescue short lifespan by these different miRNAs. Downregulation of *garz* in adult glial cells also leads to significant impairment of fly motor functions. The authors expanded their characterisation of *garz-RNAi* overexpressor flies further to show accumulation of Ref(2)P and likely consequent alterations/enlargement of mitochondria.

 Overall this is a detailed and exhaustive study. The algorithm is clearly explained and well presented, and will certainly be useful in other miRNA studies and will inspire its adaptation to other miRNA screens. Moreover the authors developed a valuable list of genes that when down-regulated in glia impact lifespan. This is a really significant resource and dataset that the authors present and should be commended for. I only have a few minor points:

 Were the longevity analysis done using males or females flies?

 Why do the authors think that some of their predictions actually resulted in lifespan extension rather than shortening? Could the down-regulation of given gene have different outcomes when it occurs in concert with other miRNA gene target downregulation? Could this be commented in discussion perhaps?

 How easily can their method be adapted for a different screen using a different output, such as for instance miRNA screen for stress resilience?

 In the ageing field, lifespan extension is a gold standard to detect anti-ageing genes and interventions. Could the authors comment on finding genes that extend lifespan in glia rather than shorten it?

 The authors say” Recently, it has been shown that siRNA knockdown of GBF1 causes intracellular APP accumulation in primary cortical neurons” , could they please define APP.

   

 In Figure 1 and 2 driver names are not very visible, could this be improved for clarity or genotypes be written in full perhaps?

 In Figure 3A, it is not very visible what the arrows is pointing at, at least not in my downloaded version of the article.

 Overall, this is a very valuable resource for anyone working on miRNA and glial cell. This research is an excellent example how screens in invertebrate organisms can lead to discoveries of important biological functions of mammalian orthologues.

Is the rationale for developing the new method (or application) clearly explained?

Yes

Are the 3Rs implications of the work described accurately?

Yes

Is the description of the method technically sound?

Yes

If applicable, is the statistical analysis and its interpretation appropriate?

Yes

Are the conclusions about the method and its performance adequately supported by the findings presented in the article?

Yes

If any results are presented, are all the source data underlying the results available to ensure full reproducibility?

Yes

Are a suitable application and appropriate end-users identified?

Yes

Are sufficient details provided to allow replication of the method development and its use by others?

Yes

Reviewer Expertise:

ageing, mTOR, autophagy, Drosophila

I confirm that I have read this submission and believe that I have an appropriate level of expertise to confirm that it is of an acceptable scientific standard.

# Response

**Published:** 2020-07-18

**Authors:** Manolis Fanto

**Affiliations:** King's College London, UK

We thank both reviewer for their competent assessment of our manuscript. We have now provided a revised version of our paper and reply here to the questions and the issues raised by both reviewers.

*1) Were the longevity analysis done using males or females flies?*

 We apologize for not having made this clear enough but lifespan analysis was done on equal numbers of males and females. This has been stressed in the revised Methods

*2) Why do the authors think that some of their predictions actually resulted in lifespan extension rather than shortening? Could the down-regulation of given gene have different outcomes when it occurs in concert with other miRNA gene target downregulation? Could this be commented in discussion perhaps?*

 While an additive effect cannot be ruled out. All verifications were done using siRNAs against single genes, the fact that some resulted in the expected phenotype and others in the opposite phenotype than that expected is probably just due to these genes not being really targeted by the miRNA that originate the prediction, or by the fact that the effect of the miRNA is predominantly recapitulated by one of the other targets.

*3) How easily can their method be adapted for a different screen using a different output, such as for instance miRNA screen for stress resilience?*

 We believe that this method can be applied to different outputs, provided that the appropriate UAS/Gal4 combination of lines and screen methods are used.

*4) In the ageing field, lifespan extension is a gold standard to detect anti-ageing genes and interventions. Could the authors comment on finding genes that extend lifespan in glia rather than shorten it?*

 We have not focused on these genes as they were not the intended scope of our work, and we also notice that in a wt background, lifespan extension is usually much milder. A possible case of interest would be *Sirt2*, which mildly extends lifespan when downregulated in our screen, but we feel it would be inappropriate to speculate on this candidate gene without a deeper investigation, like the one done here for *garz*.

*5) The authors say ”Recently, it has been shown that siRNA knockdown of GBF1 causes intracellular APP accumulation in primary cortical neurons”, could they please define APP.*

 We apologize for having overlooked this abbreviation; it is now corrected. APP is the Amyloid Precursor Protein

   

*6) In Figure 1 and 2 driver names are not very visible, could this be improved for clarity or genotypes be written in full perhaps?*

 This has now been modified in a revised Fig.1. In Fig.2 we actually do not write the name of the driver as all experiments use *repo-Gal4*, as stated in the figure legend.

*7)In Figure 3A, it is not very visible what the arrows is pointing at, at least not in my downloaded version of the article.*

 This has now been modified with some zoomed-in inset to make better visible the details pointed at by the arrow.
